# Comparison of SARS-CoV-2 spike RNA sequences in feces and nasopharynx indicates intestinal replication

**DOI:** 10.1186/s13099-022-00509-w

**Published:** 2022-08-20

**Authors:** Thomas Beck-Friis, Ambjörn Kärmander, Kristina Nyström, Hao Wang, Magnus Gisslén, Lars-Magnus Andersson, Heléne Norder

**Affiliations:** 1 Department of Infectious Diseases, Region Västra Götaland, Sahlgrenska University Hospital, Gothenburg, Sweden; 2 Department of Infectious Diseases, Institute of Biomedicine, Sahlgrenska Academy, University of Gothenburg, Gothenburg, Sweden; 3 Department of Clinical Microbiology, Sahlgrenska University Hospital, Västra Götaland Region, Gothenburg, Sweden

**Keywords:** SARS-CoV-2, COVID-19, Gastrointestinal tract, Feces, Genomic structural variation, Mutation

## Abstract

**Background:**

Little is known of possible selection and replication of SARS-CoV-2 in the intestines and if viral load in feces is associated with severity of disease. Therefore, sequence variations of the spike region in strains collected from feces and nasopharynx (NPH) from the same patients were compared. It was also investigated whether viral load in feces related to severity of COVID-19 in hospitalized patients.

**Results:**

SARS-CoV-2 RNA was found in 88 (79%) fecal samples from 112 patients. The complete spike region could be sequenced in 15 fecal and 14 NPH samples. Fourteen Alpha-variants and one Beta-variant of SARS-CoV-2 were identified. The majority of the viral genetic variants (viral populations) in two fecal samples, but none in NPH, had a reversion of the H69/V70 amino acid deletion normally seen in the Alpha variants. Nine fecal samples contained up to nine minority variants, each which may constitute a separate viral population. Five NPH samples had one genetic variant each, and one NPH sample contained nine minority populations of SARS-CoV-2 spike genes.

**Conclusions:**

The higher genomic diversity of SARS-CoV-2 in feces compared to NPH, and the reversion of the H69/V70 deletion in Alpha variants from feces indicate a selection of viral strains and replication of SARS-CoV-2 in the gastrointestinal tract.

**Supplementary Information:**

The online version contains supplementary material available at 10.1186/s13099-022-00509-w.

## Background

Individuals hospitalized due to COVID-19 usually present primarily with respiratory symptoms such as shortness of breath, oxygen desaturation and cough. However, many other symptoms are commonly found, including fever, malaise, joint pain, headache and gastrointestinal symptoms. Quantitative reverse transcription PCR (RT-qPCR) of nasopharyngeal (NPH) swabs is the most widely-used method for detection of SARS-CoV-2. A meta-analysis by Wong et al. [1] found that a significant proportion of infected patients had positive SARS-CoV-2 RNA detected in their fecal specimens. Most studies on SARS-CoV-2 genetic divergences have focused on sequencing strains from NPH samples [2–7] and few studies have sequenced the SARS-CoV-2 genomes in stool samples from patients with COVID-19 [8–12]. A question remains if the viral RNA detected in stool samples is from replicating viruses in the intestines or from viruses having replicated in the respiratory tract. We therefore wanted to study SARS-CoV-2 genomes by next generation sequencing in both NPH and fecal samples to further understand the relevance of viral RNA in feces. Patients with moderate to severe disease were sampled to compare the viral load and viral sequences of the spike region in fecal and NPH samples. The spike region was selected since it has been shown to have the highest number of amino acid changes and one of the highest mutation frequencies of all genomic regions of SARS-CoV-2 [5] The aim was to investigate if there was a correlation between the viral load and severity of disease of the patient, and if there were differences between viral sequences obtained from the two sampling sites, which could indicate compartmentalized replication with specific selections of viral strains depending on replication site.

## Results

### Factors associated with severity of disease in patients with COVID-19

In 88 of the 112 (79%) patients SARS-CoV-2 could be detected in fecal samples.

Clinical characteristics in relation to viral load are presented in Table 1. Advanced age, systemic inflammation (high CRP-levels), and lymphocytopenia were associated with a more severe clinical outcome (data not shown). Fifty-five patients had moderate disease, 33 patients had severe disease, and 24 patients, of whom 8 were intubated, had critical disease requiring intensive care, as defined by the WHO Clinical Progression scale [13].

**Table 1:** Demographic factors related to the viral load of SARS-CoV-2 in feces (*N* = 88)

Patient factor	Viral load^v^			*P*-value
	Low *N* = 22 (%)	Moderate *N* = 38 (%)	High *N* = 28 (%)	
Male gender^a^	15 (68)	25 (66)	16 (57)	0.5^c^
Age^b^	59 ± 15	59 ± 13	60 ± 15	0.9^d^
Critical disease^a^	4 (18)	11 (29)	5 (18)	0.1^c^
Severe disease^a^	7 (32)	12 (32)	5 (18)	0.2^c^
Days spent in hospital^b^	14 ± 15	17 ± 18	17 ± 22	0.9^d^
Days of symptoms prior to stool sampling^b^	14 ± 5	12 ± 4	11 ± 4	0.09^d^
Thirty-day mortality^a^	1 (5)	2 (5)	0 (0)	0.7^c^
CRP maximum (mg/L)^b^	160 ± 70	160 ± 80	130 ± 90	0.4^d^
Lymphocytes minimum (10^9^/L)^b^	0.8 ± 0.3	0.9 ± 0.4	0.9 ± 0.3	0.7^d^
Body mass index (kg/m^2^)^b^	30 ± 5	30 ± 7	29 ± 5	0.9^d^
Hypertension^a^	7 (32)	15 (39)	10 (36)	0.6^c^
Other morbidity^af^	9 (41)	7 (18)	13 (46)	0.07^c^
Immunosuppression^a^	1 (5)	1 (3)	2 (7)	0.8^c^
Remdesivir treatment^a^	1 (5)	2 (5)	2 (7)	1^c^
Corticosteroid treatment^a^	17 (77)	34 (89)	19 (68)	0.2^c^
IL6 receptor-inhibitor treatment^a^	3 (14)	2 (5)	2 (7)	0.7^c^

^v^Negative (cycle threshold > 39); low (Ct 36–39); moderate (Ct 32–36); high Ct < 32)

^a^Sum (%)

^b^Mean value ± standard deviation

^c^Pearson Chi-square

^d^One-way ANOVA

^f^Diabetes mellitus type 1 or 2, chronic lung disease, chronic kidney disease and/or malignancy

**Fig. 1:**
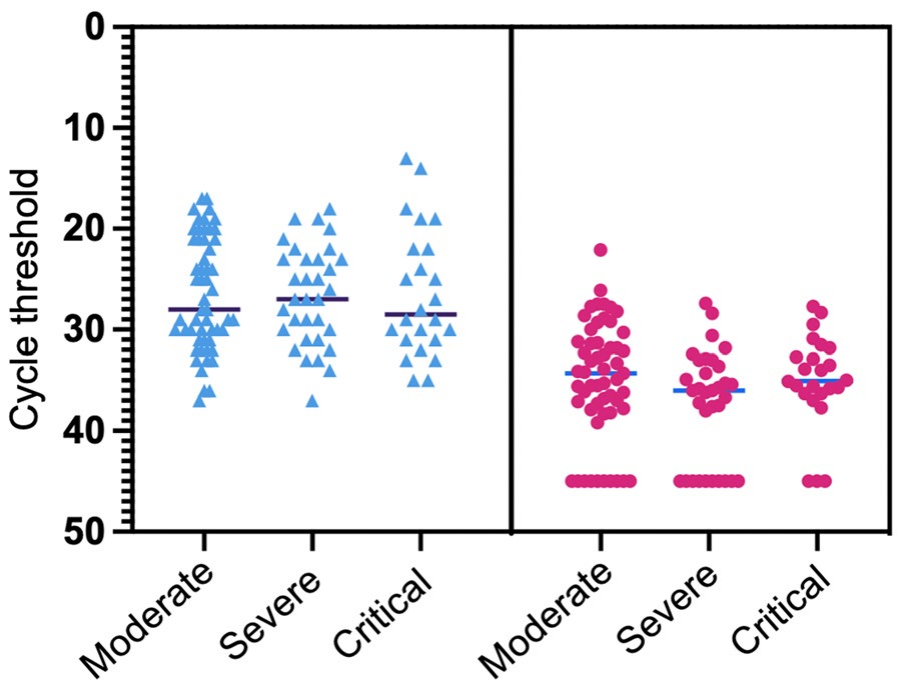
Relationship between cycle threshold (Ct) values and severity of disease. Ct values, with a maximum of 45 cycles, were derived from PCR analyses for SARS-CoV-2 RNA in nasopharyngeal (blue triangles) and stool (red circles) samples acquired from 112 hospitalized patients with COVID-19 from March 2021 through May 2021. Severity of disease was categorized as Moderate (N = 55, non-High Flow Nasal Oxygen (HFNO)-dependent), Severe (N = 33, HFNO-dependent), and Critical (N = 24, Intensive Care-dependent) (13) Lines represent median

As shown in Fig. 1, the Ct values of SARS-CoV-2 RNA in fecal and NPH samples did not differ significantly between severity-groups. The Ct values for SARS-CoV-2 in NPH were, however, significantly lower than those in the fecal samples (p < 0.001), corresponding to higher viral load in the NPH. Median [IQR] days of symptoms before sampling were significantly higher for fecal samples (11 [5]) compared with NPH samples (9.5 [5]), (p < 0.001).

### Next generation sequencing of SARS-CoV-2 in feces and NPH

The qPCR Ct values for SARS-CoV-2 were, on average, 33.4 ± 3.4 (mean ± SD) in the 88 fecal samples. Sequencing was attempted in strains from 29 qPCR-positive fecal samples with a Ct value ≤ 32 (n = 29; 33%) for SARS-CoV-2. The complete spike genomic region, known to more frequently mutate than other regions of the genome [5], was obtained for the strains in 15 of these fecal samples, and the NPH sample of corresponding patients, although one NPH sample was not recovered (patient 3). For another 9 fecal samples partial spike region sequences were obtained. On average, Ct values for SARS-CoV-2 in the fecal samples with a complete spike genomic region sequence were 29.8 ± 2.1 and the Ct values of SARS-CoV-2 in the 14 corresponding NPH samples were 24 ± 5. Median [IQR] days between collection of NPH- and fecal samples was 1 [4]. The Alpha variant of SARS-CoV-2 was identified in the samples from 14 of the patients, whereas patient 9 was infected with the Beta variant. During the study period Alpha was the dominant variant (79%) in Sweden [14].

**Fig. 2:**
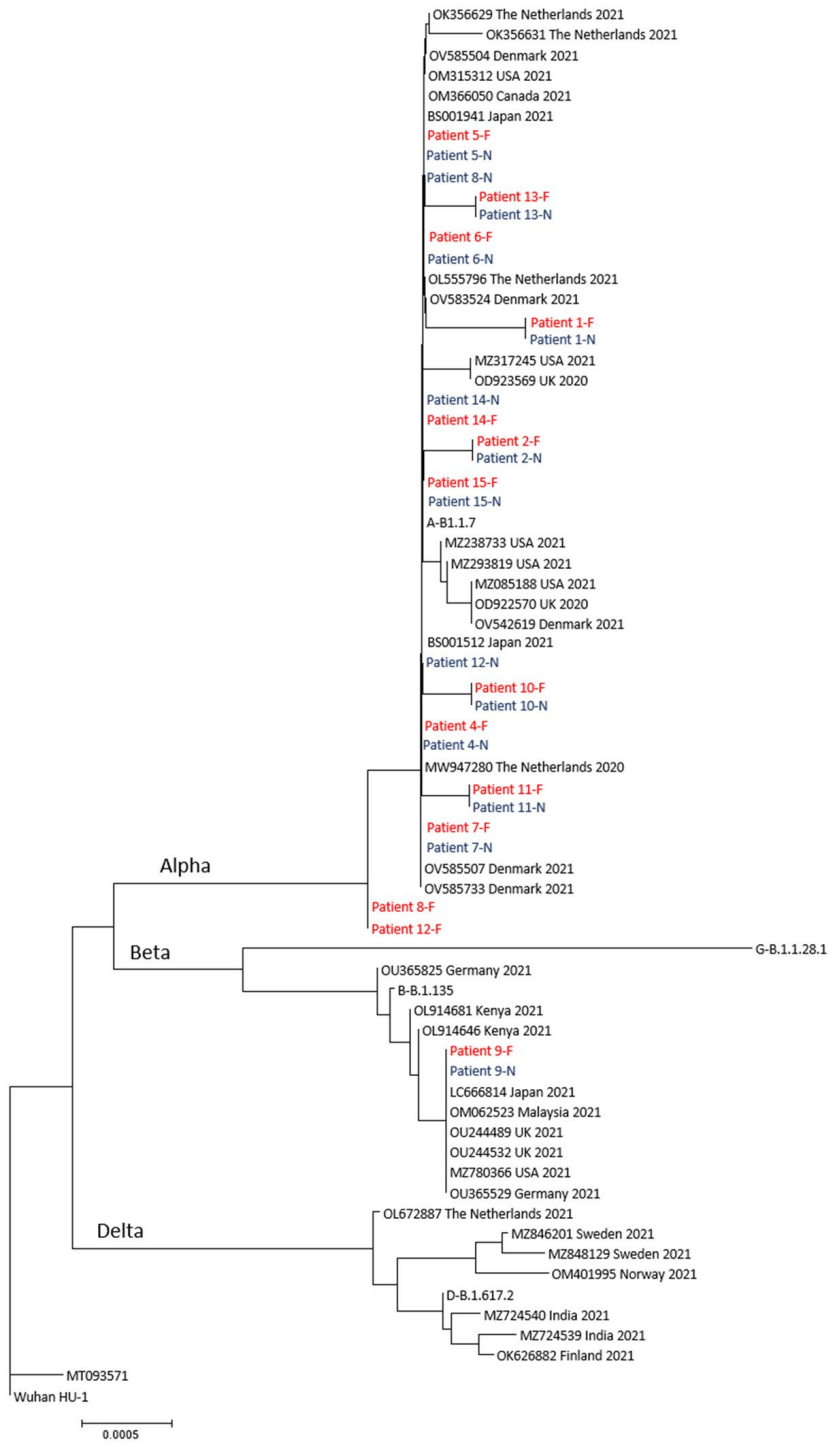
Neighbor joining tree based on 3822 nucleotides of SARS-CoV-2 spike protein gene of consensus sequences from fecal samples, NPH-samples and selected sequences from Genbank. Strains are named with Genbank accession number, country of origin and year of collection. *F* fecal sample (red); *N* NPH sample (blue)

As shown in Fig. 2, SARS-CoV-2 strains from 12 of the patients had identical consensus sequences of the spike region in the NPH- and fecal samples. The amino acid H69/V70 deletion, normally seen in Alpha variant, was not present in the fecal samples from patients 8 and 12, but was present in the NPH samples from these patients. The Alpha variant infecting four patients each had one nucleotide substitution in the consensus sequence of the spike protein gene in strains both from NPH and feces, one of these patients had an Alpha variant whose nucleotide substitution was non-synonymous [15], resulting in an amino acid change. Another Alpha variant from patient 1 had two nucleotide substitutions, both non-synonymous, compared to the Alpha reference strain accession number MZ344997 (Table 2). Strains from patient 9, the only patient infected with the Beta variant, had one nucleotide substitution that was non-synonymous, compared to the Beta variant reference strain accession number MW598419 (Table 2).

**Table 2:** Changes in the SARS-CoV-2 spike protein gene in the feces and the nasopharynx in patients hospitalized with COVID-19 (N = 14)

Pat-ient	Feces			NPH			Comparison feces vs NPH		Days of symptoms prior to fecal sample	Days from NPH sample to fecal sample
	Popula-tions (%)	Number changes majority population^a^	Number changes minority population^b^	Popula-tions (%)	Number changes majority population^a^	Number changes minority population^b^	Difference between majority populations	Difference between minority populations		
1	81/19	2	6	92/8	2	1	0	5	8	2
2	91/9	1	5	90/10	1	1^c^	0	6	11	1
4	94/6	0	3^c^	100	0		0	x	15	4
5	100	0		100	0		0		9	1
6	100	0		100	0		0		5	4
7	94/6	0	1	100	0		0	x	9	−3
8	83/17	1^c^	9	91/9	0	1^c^	1	10	8	0
9	95/5	1	1	100	1		0	x	17	4
10	100	1		89/11	1	1	0	y	7	0
11	94/6	1	1	91/9	1	1^c^	0	2	8	8
12	91/9	1^c^	4	100	0		1	x	7	1
13	100	1		100	1		0		10	8
14	100	0		90/10	0	9	0	y	13	−5
15	94/6	0	2	100	0		0	x	12	−1

^a^The spike RNA sequence of the Alpha variant (GenBank accession number: MZ344997.1) is used as reference except for patient 9 where the spike RNA sequence of the Beta variant (GenBank accession number: MW598419.1) is used as a reference

^b^The majority population of each corresponding sample is used as reference

^c^Reversion of amino acid 69/70 deletion present

x = viral minority populations only found in fecal samples

y = viral minority populations only found in NPH samples

Patient 3 not shown as the NPH sample was not recovered

**Fig. 3:**
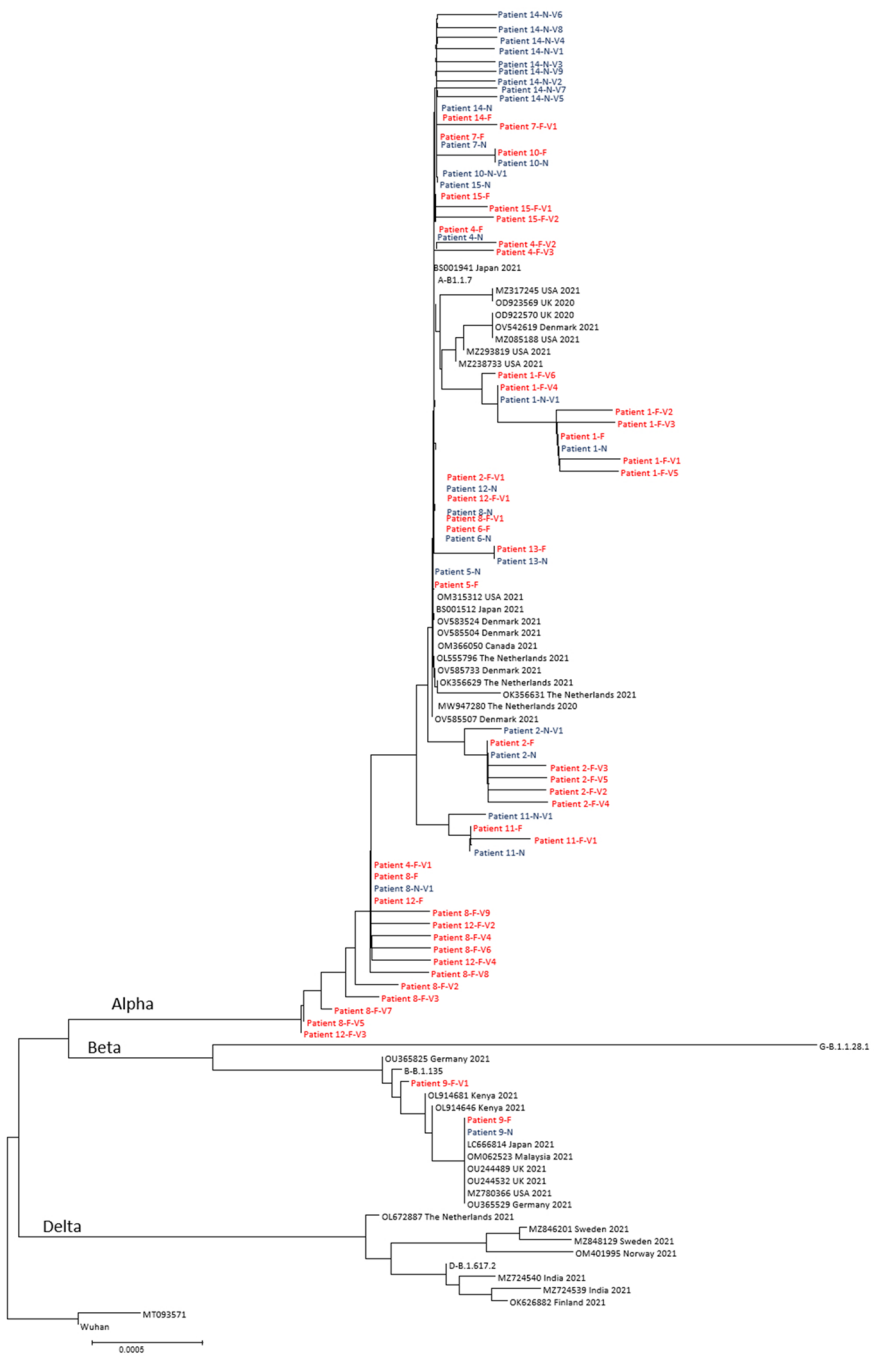
Neighbor joining tree based on 3822 nucleotides of SARS-CoV-2 spike protein gene with consensus sequences and variants from fecal samples (red color) and NPH-samples (blue color). The tree also includes selected sequences from GenBank, designated with accession number, country of origin and year of collection. Each variant has one change that differs from the consensus sequence. *F* fecal sample, *N* NPH sample. Variants are named with *V1* variant 1, *V2* variant 2 etc.

Between one to nine different SARS-CoV-2 minority populations were found in strains from nine fecal and six NPH samples (Fig. 3 and Table 2). Minority populations were found in strains from samples collected from 11 of the 14 patients.

For five patients, minority populations were only found in strains from feces and not in NPH, while samples from two patients’ minority populations were only found in strains from NPH. The number of minority populations of strains found in all fecal samples were larger than those found in NPH (Table 2). The different variants in the fecal samples were found in up to 39% of the sequences. Most, 82.8%, of the nucleotide substitutions of the variants were non-synonymous, resulting in 4 amino acid changes in the N-terminal domain, 4 amino acid changes in the receptor binding domain and 12 amino acid changes in the S2 region of the spike protein.

Strains from three NPH samples also had minority populations differing from the corresponding majority population only by reversion of the H69/V70 deletion. Two of the other three strains from NPH had minority populations with only one nucleotide substitution (Table 2). The strain from the sixth NPH sample (patient 14) was an outlier with nine base substitutions in the minority population. This patient also differed clinically as the only patient in this study in need of a ventilator, of the sequenced samples, and who had detectable SARS-CoV-2 RNA in NPH for approximately 1.5 months. NPH samples had an average of 1.8 base substitutions per sample with a minority population (0.4 base substitutions when patient 14 was omitted) with frequencies up to 19%, where 72.7% were non-synonymous (50% non-synonymous when patient 14 was omitted).

Except for reversions to wild type, only one nucleotide substitution, C2042A found in 5% of the sequences in the fecal minority population of patient 9, infected with the Beta variant, was shared with all the sequences from the other patients’ samples, as this substitution is normally present in the SARS-CoV-2 Alpha variant.

As can be seen in Fig. 3, the different variants from each patient sample formed clades in the phylogenetic tree. Most variants obtained from both NPH and fecal samples were similar. There are, however, exceptions, as can be seen in the clades formed by variants in samples from patients 2, 4, 8, 11 and 12, due to the H69/V70 deletion. Other than the H69/V70 deletion, all mutations are nucleotide substitutions and a minority of these substitutions are reversions to wild type SARS-CoV-2 of mutations present in the Alpha or Beta variant.

## Discussion

This study showed a greater viral genomic diversity of SARS-CoV-2 in fecal compared to NPH samples from hospitalized patients with COVID-19. We found that two consensus strains and one minority strain out of 13 Alpha variants of SARS-CoV-2 had reverted the Alpha specific H69/V70 deletion [16] in the fecal samples, although it was present in NPH. In addition, there was a greater variety of virus strains in feces compared to NPH. Both the reversion of H69/V70 deletion in the SARS-CoV-2 genomes in the fecal viral populations as well as the emergence of new mutations in the minority populations are possibly explained by ongoing replication in both the respiratory tract and the gastrointestinal tract causing a divergence between the two populations. So far, studies have suggested intestinal viral replication of SARS-CoV-2 [17], with virus identified in feces, known expression of ACE2 in the intestinal tract and replication of the virus in intestinal organoids. However, little data is available regarding replication of SARS-CoV-2 in human intestine. We believe that our data adds to this knowledge, and indicates that intestinal replication and furthermore local intestinal selection of SARS-CoV-2 strains is occurring.

There have been conflicting reports on whether patients with PCR-positive rectal swabs or stool samples for SARS-CoV-2 have a higher risk for a more severe clinical course of COVID-19 [18, 19]. However, two other studies [20, 21] found that there was no increased risk for severe disease in patients with PCR-positive stool samples. In the present study, stool sample positivity rate did not correlate with severity of disease. The patients with severe and critical disease were sampled at a later stage of the disease course (p < 0.001) than the patients with moderate disease, which makes it difficult to evaluate a possible correlation without taking symptom duration into account. As also previously shown, advanced age, systemic inflammation, and lymphopenia were significantly linked to a more severe clinical course [22].

The clinical utility of detection of SARS-CoV-2 RNA in stool samples from patients hospitalized with COVID-19 appears to be low, as severe disease cannot be prognosticated from stool sample results and the viral load was lower in stool samples compared to NPH samples. The detection rate could probably be increased by collecting stool samples rather than swabs, as done in this study. Typing may, however, be of importance if prolonged shedding of the virus would induce selection of different strains in feces compared to NPH.

Relative to what has been published to date, our study has strength in numbers and percentage of patients with SARS-CoV-2 RNA in feces [8–12]. However, this study has several limitations. The patients were not sampled with paired fecal and NPH samples on the same day and different methods were used to quantify viral load in the two different sample types, which could have had impact on the positivity rates and comparisons of viral loads in NPH and feces. Also, the duration from onset of symptoms to sampling was considerably longer for patients with severe and critical disease, which could impact positivity rates and viral load comparisons between patients with different severity of COVID-19.

Most known human fecal/orally transmitted viruses such as calici, rota, astro, adeno and enteroviruses are non-enveloped making them less susceptible to the harsh environment of the digestive system [23]. SARS-CoV-2 is an enveloped virus and should therefore not be able to remain infectious in the gastrointestinal tract. However, four different coronaviruses can infect the gastrointestinal tract in pigs [24] and the enveloped avian influenza virus reproduces in the intestines [25]. The possibility for infection in humans by enveloped viruses should therefore not be dismissed solely based on the viral structure. One possible explanation for how SARS-CoV-2 infects the gastrointestinal tract is by haematogenous spreading [17, 26]. However, ACE2, the viral receptor expressed in various different human tissues [27], is mainly expressed on the apical surfaces of the enterocytes, which suggests that the cells are exposed for infection mainly from the intestinal lumen [27–29]. Further, Zang et al. found that SARS-CoV-2 mainly infects the apical surface of human intestinal epithelial cells in vitro [30]. After a primary infection in the respiratory tract is established, virions may also be swallowed down together with saliva and mucus. This could act as a protective layer shielding the virions from the gastric acid as well as bile salts and pancreatic juices. This has been shown for both influenza virus A and B viruses, which when mixed with highly viscous artificial mucus remained infectious after several hours in environments simulating bile, pancreatic juices and gastric acids [31]. Amino acid alterations in receptor regions may change viral pH-resistance as is shown for avian influenza A [32] and viral tropism from intestinal to respiratory replication of the virus as is shown for porcine respiratory coronavirus [33]. The amino acids H69/V70 deletion was present in the spike protein of SARS-CoV-2 virions in NPH, as these patients were infected with the alpha variant, but in two patients this deletion had reverted in the majority of spike sequences detected in feces. The role if this specific deletion has been shown to increase the infectivity of the virus in Hek293T and Calu-3 cell lines [16] as well as be important for neutralizing antibodies [34]. Our data indicates selection of specific viruses in the intestinal tract, but the role of these amino acid residues in intestinal infection of SARS-CoV-2 requires further investigation. Also, the higher genomic diversity of SARS-CoV-2 spike gene in feces compared to NPH found in this study needs further understanding. This appears to occur regardless of the severity of the disease and may be important for intestinal replication of SARS-CoV-2. The selection of virus strains in the gastrointestinal tract during infection identified in this study may also have consequences for the spread and evolution of the virus and should be further investigated.

## Conclusion

Next Generation Sequencing of SARS-CoV-2 RNA in fecal- and NPH samples showed a greater number of sequence variantion in feces as compared to corresponding NPH as well as a reversion to wild type of the H69/V70 deletion in two out of 13 Alpha variants in fecal samples but not in NPH samples. This can possibly be explained by ongoing replication in the intestinal- and the respiratory tract. An increased understanding of SARS-CoV-2 as an intestinal pathogen can have implications for public policy making and for finding effective cures.

## Methods

Fecal samples were collected from 112 patients (74:38 [male: female]; 60 ± 14 [age ± SD] years) who were hospitalized with COVID-19 and admitted to the Department of Infectious Diseases, Sahlgrenska University Hospital, Gothenburg, Sweden from March through May 2021. The study was a sub-study of an ongoing prospective COVID-19 cohort study [35, 36]. All patients were hospitalized and had laboratory-confirmed COVID-19 determined by RT-qPCR for SARS-CoV-2 from nasopharyngeal swabs. The first day of symptoms was defined by the first day of at least one of the following symptoms prior to hospitalization: fever or chills, cough, shortness of breath or difficulty breathing, fatigue, muscle or body aches, headache, new loss of taste or smell, sore throat, congestion or runny nose, nausea or vomiting, or diarrhea [37].

Patients were divided into three groups depending on the severity of disease: moderate–hospitalized, but not high flow nasal oxygen (HFNO)-dependent; severe–HFNO-dependent; and Critical–Intensive care-dependent [13]. Clinical data and results from routine laboratory tests were retrieved from medical records and recorded in a case report form.

Feces was collected in tubes at the hospital ward and stored at 4 °C before they were transferred to the laboratory, the samples were stored at – 80 °C if not extracted upon arrival.

### RNA extraction and quantification

RNA was extracted from the feces using the AllPrep PowerViral DNA/RNA Kit (Qiagen, Germany). Approximately 0.25 g of feces was added to 600 µl of the lysis buffer supplied in the kit and was homogenized at 3000 RPM in Minilys (Bertin instruments, France) for 4 min. After homogenization, the manufacturer’s protocol was followed and nucleic acids were eluted in 100 µl RNase free water. All samples were analyzed for SARS-CoV-2 RNA with qPCR. Nucleic acids in samples with cycle threshold (Ct) values ≤ 32 were also extracted with NucliSENS easyMAG (bioMérieux, France) as sequencing quality was improved when sufficient SARS-CoV-2 RNA could be extracted. Approximately 0.25 g of feces was transferred to a 10 ml tube with 2 ml PBS-buffer and glass beads. The material was then homogenized by vortexing until the solids in the feces had dissolved. The tube was centrifuged for 5 min at 3000 g, 250 µl of the supernatant was then extracted using the manufacturers off board lysis protocol. The elution volume was 110 µl.

Real-time quantitative PCR was performed using the Applied Biosystems 7300 System (ThermoFisher Scientific, Massachusetts, USA) as described previously [38] with the modification that the mastermix contained 40 U RNaseOUT (ThermoFisher Scientific, Massachusetts, USA) and one dilution (1/10^6^) of 2 µg/ml plasmid (Eurofins genomics, Luxembourg) was used as a positive control.

Quantification of SARS-CoV-2 in nasopharyngeal samples had been determined by using either the cobas^©^ SARS-CoV-2 test on the cobas^©^ 6800 system (Roche Diagnostics, Switzerland) or by using the Xpert Xpress SARS-CoV-2/Flu/RSV kit on the GeneXpert IV system (Cepheid, California, USA). Nasopharyngeal samples that underwent sequencing were extracted by using the MagNA Pure 96 DNA and Viral NA Small Volume Kit on the MagNA Pure 96 System (Roche Diagnostics, Switzerland).

### Next generation sequencing

SARS-CoV-2 RNA, extracted from the fecal samples with a Ct value below or equal to 32 (n = 29) were sequenced, using Ion Torrent (ThermoFisher Scientific, Massachusetts, USA) as described previously [39].

SARS-CoV-2 RNA in nasopharyngeal samples was sequenced, using either Ion Torrent [39] or Illumina (California, USA). When using Illumina, the Illumina COVIDSeq test kit was used in accordance with the protocol from the manufacturer (llumina, California, USA). Briefly, cDNA was constructed from RNA extracted with MagnaPure 96 by using random hexamer primers. Samples were amplified with COVIDSeq Primer Pool 1 HT and COVIDSeq Primer Pool 2 HT primers (llumina, California, USA). Amplified cDNA was fragmented, tagged with adaptor sequences, amplified and pooled using the automated Tecan Fluent 780 system (Tecan, Männedorf, Switzerland). The pooled libraries were quality checked, quantified and sequenced using the NextSeq 500 system with Mid Output kit V2 (300 c) or the Novaseq 6000 system with S2 reagent kit (300 c).

Assembly of consensus sequences and the determination of SARS-CoV-2 lineages in samples sequenced with Ion Torrent were made with IRMAreport plug-in (ThermoFisher Scientific, Massachusetts, USA) and assigned to variants, using Pangolin methods [39]. For strains in samples sequenced with Illumina, assembly and determination of SARS-CoV-2 lineages were made with the Arctic Networks nCoV-2019 novel coronavirus bioinformatics protocol running on a nextflow pipeline developed by Connor Lab [40]. CLC Genomic Workbench 12 (Qiagen, Hilden, Germany) was used for variant calling, minimum frequency was set to 5% and minimum depth was set to 100 reads. GenBank accession numbers for the consensus sequences of the spike region are presented in Additional file 1: Table S1.

### Phylogenetic analysis

The complete spike gene, 3822 nucleotides, obtained from strains in fecal and NPH samples from 14 patients were aligned with 39 sequences of the corresponding region obtained from GenBank. The sequences from GenBank were selected to represent different strains of Alpha, Beta or Delta variants from various countries. The evolutionary distance was calculated using Maximum Composite Likelihood method by using a gamma distribution with alpha 0.03 in the program Mega6 and phylogenetic trees were constructed using neighbor-joining (NJ) method [41].

### Statistical analysis

Continuous variables were compared using one-way ANOVA-test and comparisons of proportions were made using Pearson’s Chi-squared or Fischer’s exact tests as appropriate. P values < 0.05 were considered statistically significant. All statistical analyses were done with the SPSS software package version 27.0.0.0 (IBM, Armonk, New York, US).

## Supplementary Information

**Additional file 1:** **Table S1.** GenBank accession numbers for the consensus sequences of the fecal- and nasopharyngeal samples.

## Data Availability

GenBank accession numbers for the consensus sequences of the spike region are presented in Additional file 1: Table S1.
