# Supplementary material for: Comparison of SARS-CoV-2 spike RNA sequences in feces and nasopharynx indicates intestinal replication
Source: Gut Pathog. 2022 Aug 20;14:35. doi: 10.1186/s13099-022-00509-w (PMC9392503; doi:10.1186/s13099-022-00509-w)
Supplement: Supplementary file 1 — Additional file 1: Table S1. GenBank accession numbers for the consensus sequences of the fecal- and nasopharyngeal samples. [file 13099_2022_509_MOESM1_ESM.pdf]

Patient	Type of material	GenBank Acession number
1	Feces	ON080526
	NPH	ON080527
2	Feces	ON080528
	NPH	ON080529
4	Feces	ON080530
	NPH	ON080531
5	Feces	ON080532
	NPH	ON080533
6	Feces	ON080534
	NPH	ON080535
7	Feces	ON080536
	NPH	ON080537
8	Feces	ON080538
	NPH	ON080539
9	Feces	ON080540
	NPH	ON080541
10	Feces	ON080542
	NPH	ON080543
11	Feces	ON080544
	NPH	ON080545
12	Feces	ON080546
	NPH	ON080547
13	Feces	ON080548
	NPH	ON080549
14	Feces	ON080550
	NPH	ON080551
15	Feces	ON080552
	NPH	ON080553

Supplementary Table 1. GenBank accession numbers for the consensus sequences of the fecal- and nasopharyngeal samples.
